# Under-reporting of TB cases and associated factors: a case study in China

**DOI:** 10.1186/s12889-019-8009-1

**Published:** 2019-12-11

**Authors:** Danju Zhou, Michelle Pender, Weixi Jiang, Wenhui Mao, Shenglan Tang

**Affiliations:** 1 Duke Global Health Institute, Duke University, Durham, NC USA; 2 Duke Kunshan University, Kunshan, China; 3 SingHealth Duke-NUS Global Health Institute, Singapore, Singapore

**Keywords:** Tuberculosis, TB reporting, Health information management systems, Human resource management, China

## Abstract

**Background:**

Tuberculosis is a leading cause of death worldwide and has become a high global health priority. Accurate country level surveillance is critical to ending the pandemic. Effective routine reporting systems which track the course of the epidemic are vital in addressing TB. China, which has the third largest TB epidemic in the world and has developed a reporting system to help with the control and prevention of TB, this study examined its effectiveness in Eastern China.

**Methods:**

The number of TB cases reported internally in two hospitals in Eastern China were compared to the number TB cases reported by these same hospitals in the national reporting systems in order to assess the accuracy of reporting. Qualitative data from interviews with key health officials and researcher experience using the TB reporting systems were used to identify factors affecting the accuracy of TB cases being reported in the national systems.

**Results:**

This study found that over a quarter of TB cases recorded in the internal hospital records were not entered into the national TB reporting systems, leading to an under representation of national TB cases. Factors associated with underreporting included unqualified and overworked health personnel, poor supervision and accountability at local and national levels, and a complicated incohesive health information management system.

**Conclusions:**

This study demonstrates that TB in Eastern China is being underreported. Given that Eastern China is a developed province, one could assume similar problems may be found in other parts of China with fewer resources as well as many low- and middle-income countries. Having an accurate account of the number of national TB cases is essential to understanding the national and global burden of the disease and in managing TB prevention and control efforts. As such, factors associated with underreporting need to be addressed in order to reduce underreporting.

## Background

Tuberculosis (TB) is one of the top ten causes of death worldwide, and the leading cause of mortality from a single infectious agent (1). In 2017 alone, ten million people contracted TB and 1.6 million people died (2) . Ending the TB pandemic has become a global health priority, and an important part of the Sustainable Development Goals (SDGs). The World Health Organization (WHO) *End TB Strategy* calls for an 80% reduction in the TB incidence rate and 90% reduction in the number of TB deaths between 2015 and 2030 (1). The strategy calls for “government stewardship and accountability with monitoring and evaluation” and “eliciting full benefits of health and development policies and systems” (1) in order to achieve these goals. Having an accurate account of the number of TB cases in any country is critically important to estimate the national and global burden of TB, and to calculate the necessary resources required for each country to combat the epidemic now and in the future.

China has the third largest TB epidemic in the world with an estimated one million new cases every year (3), and therefore plays an important role in the efforts to end the TB pandemic. After the severe acute respiratory syndrome (SARS) outbreak demonstrated the limitations of China’s infectious disease surveillance system, the government established a standardized nationwide system known as the National Notifiable Disease Reporting System (NNDRS). The NNDRS is a surveillance system used by all health facilities in China to provide information on 39 notifiable infectious diseases, including TB, within 24 h of diagnosis (4, 5). In addition, a TB specific surveillance system known as the TB Information Management System (TBIMS) was established as a platform for all health facilities to report confirmed TB cases as well as additional patient information within 48 h (5). In order to enter information in TBIMS, patient information is first recorded in the Hospital Information System (HIS) and in NNDRS by staff responsible for reporting TB cases at the individual hospitals. After the TB cases have been confirmed in the patients, the staff are then required to enter information from NNDRS into TBIMS (6). However, previous studies have found that these reporting systems require improvement (7–14), and one study found that the new surveillance systems have not lead to a significant decrease in the number of unreported cases (7). In particular it was found that the surveillance systems fail to capture all TB cases in situations where health workers using the systems are poorly trained and incentivized, and where there is ineffective communication between hospitals and the Centers for Disease Control and Prevention (CDC) (11, 12). Given the importance of accurate reporting, WHO has spearheaded a global effort to strengthen reporting systems (15). India and Indonesia have undertaken efforts to test the accuracy of their reporting systems to help address their high rates of TB (16, 17). Some research has examined the reporting systems in China. Wubuli and her colleagues noted that in Xinjiang Province, TB cases might be missed in routine notification systems due to the fact that private providers without authorization to diagnose TB often do so anyway but usually do not report these cases to the local CDC (18). Research on other infectious diseases in China found prevalence of underreporting. Chen et al. found that brucellosis was underreported in Shanxi Province (19). In addition, He et al. discussed the potential underreporting problems of HBV in Zhejiang Province and found that only 17.85% of HBV cases reported in NNDRS included personal ID numbers, which increased the possibility of underreporting (20). However, there is still insufficient rigorous research on the extent of underreporting of TB in China (13, 14).

The aim of this paper is to empirically analyze the issues of TB underreporting, examine the reliability of the health information systems established to track TB in China, and analyze the main factors associated with underreporting.

## Methods

The study analyzed reported TB cases in hospital information systems and TB information management systems from two hospitals to determine if any TB cases were unreported. All research was conducted in City P,^1^ located in a developed region of Eastern China. City P has an established and integrated health information system, including HIS, NNDRS and TBIMS. This study employed mixed methods to collect quantitative data from health information system records and qualitative data from semi-structured interviews.

Quantitative data included medical records collected from two TB designated hospitals in City P (Hospital A and Hospital B), and TBIMS records for City P. City P includes three counties with similar socio-economic levels, and each of these counties has one designated TB hospital. Data was also collected from a third hospital in the third county, however, this data was of poor quality and lacked the key variables collected by the other two hospitals in the study. Therefore, we were unable to sufficiently include the third hospital data in our comparison and analysis. The data used for this study are not publicly available and so permission was obtained to access the data from the local health commission. HIS records dated between January 1st, 2015 and December 31st, 2017 were included for analysis. Hospital A included both outpatient and inpatient records, Hospital B’s inpatient records were the only reliable records available and so the study did not include the outpatient records. Records in which the diagnosis contained the word “tuberculosis”, or where the sputum smear and culture results were positive were included in the study (21). Cases with suspected TB; Extra-pulmonary TB; and Old TB, or where the diagnosis was marked as “?”, “to be checked” or “to be excluded” and duplicate records were excluded from the study. TBIMS records from January 1st, 2012 and December 31st, 2017 were included. The time period for TBIMS is longer than HIS since TB treatment requires a long regimen, and patients entered in HIS may have been reported before 2015. All confirmed TB cases recorded in each HIS were compared to the cases reported in TBIMS. Confirmed TB cases in HIS which did not also appear in TBIMS were considered as unreported. The demographic and social characteristics of the unreported TB patients were analyzed to identify possible factors leading to their exclusion. After identifying the underreported cases, a bivariable analysis was used to compare the quantitative variables for unpaired data. For categorical indicators such as age group, address, and discharge department, we performed a chi-square test to evaluate how TB patients’ specific characteristics differed between the reported and unreported cases. A *p*-value of less than 0.05 was considered statistically significant.

Qualitative data was collected through 13 semi-structured in-depth interviews with 5 categories of people responsible for using the TB reporting system (an English interview guide can be found in Additional file 1). Those interviewed included: the reporter responsible for CDC TBIMS, two leaders responsible for infectious diseases, three TB reporters, and four TB clinicians in the two hospitals, and the head of CDC (while the CDC do not work in the hospitals, they are responsible for verifying data entered into TBIMS and are very familiar with the reporting systems). Informed consent was obtained from all participants. In addition to these interviews, a member of the research team performed the functions of the member of staff responsible for reporting TB cases for one day. Assuming this role enabled us to more comprehensively understand the functions of reporting systems and identify places where errors may occur and improvements could be made. Qualitative data were analyzed using Nvivo software.

## Results

Our study found that 26% of TB cases were unreported in the two hospitals. The rate of unreported cases in inpatient records for Hospital A were slightly lower than inpatient records for Hospital B, 13 and 18% respectively. The highest rate of unreported cases was found in the outpatient records in Hospital A (36%) (Table 1).

**Table 1:** Overall Underreported Rate

	Confirmed TB cases which should be reported. (*N*)	Confirmed TB cases which were actually reported. (*N*)	Unreported cases (*N*)	Rate of unreported cases
Hospital A (Outpatient)	1034	658	376	36.4%
Hospital A (Inpatient)	639	554	85	13.3%
Hospital B (Inpatient*)*	234	193	41	17.5%
In Total	1907	1405	502	26.3%

The medical records included demographic characteristics such as age, sex, address, and occupation (Table 2).

**Table 2:** Socio-Demographic Characteristics of Tuberculosis Cases by Reported Status, By Bivariate Analysis

	Hospital A (*n* = 1034) TB Cases Unreported / Confirmed *Outpatient* Cases Average, or No. (%)	Hospital A (*n* = 639) TB Cases Unreported / Confirmed *Outpatient* Cases Average, or No. (%)	Hospital B (*n* = 234) TB Cases Unreported / Confirmed *Inpatient* Cases Average, or No. (%)
All Ages	47.4 / 51.2 ***	58.7 / 57.3	68.1 / 66.4
Age less than or equal to 35	131 / 281 (46.6) ***	16 /124 (12.9)	1 / 14 (7.1)
Age greater than 35 and less than or equal to 65	165 / 471 (35.0)	30 / 256 (11.7)	16 / 77 (20.8)
Age greater than 65	80 / 282 (28.4) ***	39 / 259 (15.1)	24 / 143 (16.8)
Male	258 / 731 (35.3)	66 / 465 (14.2)	34 / 179 (19.0)
Female	118 / 303 (38.9)	19 / 174 (10.9)	7 / 55 (12.7)
Has personal identity card	229 / 837 (27.4) ***	82 / 630 (13.0)	36 / 213 (16.9)
Does not have personal identity card	147 / 197 (74.6) ***	3 / 9 (33.3)	5 / 21 (23.8)
2015 admission year	305 / 660 (46.2) ***	39 / 229 (17.0)	22 / 87 (25.3) *
2016 admission year	48 / 269 (17.8) ***	27 / 204 (13.2)	6 / 70 (8.6) *
2017 admission year	23 / 105 (21.9) ***	19 / 206 (9.2)	13 / 77 (16.9)
Current address in under jurisdiction	354 / 974 (36.7)	78 / 617 (12.6) ***	40 / 229 (17.5)
Current address not under jurisdiction	22 / 60 (36.7)	7 / 22 (31.8) ***	1 / 5 (20.0)
Had in-hospital referral	N/A for outpatients	20 / 55 (36.4) ***	31 / 191 (16.2)
Did not have in-hospital referral	N/A for outpatients	65 / 584 (11.1) ***	10 / 43 (23.3)
Primary occupation farmer	Not reported	Not reported	35 / 209 (16.7)
Primary occupation not a farmer	Not reported	Not reported	6 / 25 (24.0)
Total	376 / 1034 (36.4)	85 / 639 (13.3)	41 / 234 (17.5)

Note: *P < .05; **P < .01; ***P < .001

Age and address of residence were the only demographics with any significance. Age was significant for the outpatient cases in Hospital A where the average age of patients unreported was 47, and the average age of all the cases was 51. Underreporting was significant among age group 35 and under (47%) and over age 65 (28%) outpatients in Hospital A. Address of registration was a significant factor for inpatient cases in Hospital A, patients residing in the jurisdiction of the hospital were less likely to be unreported than patients who did not reside in the jurisdiction (13 and 32% respectively). Other characteristics that proved significant were: possessing a personal identity card; the year of admission; and whether or not there was an in-hospital referral. Patients without a personal ID were much more likely go unreported than those with an ID card (75 and 27% respectively). The admission year also played a factor in reporting. Among outpatients in Hospital A, 2015 had the highest unreported rate (46%) compared to 2016 (18%) and 2017 (22%). Hospital B had the highest unreported rate among inpatients in 2015 (17%), compared to 2017 (9%), however the lower *p* values (*P* < .05) for these years give us less certainty that we can reject the null here. Finally, inpatients in Hospital A who had received referrals within the hospital were more likely to be unreported than inpatients who did not have an in-hospital referral (36 and 11% respectively).

### Health system related factors associated with the underreporting

Through interviews and an in-depth study of the current functions of the TB reporting system we identified two main areas which may cause underreporting: health information management, and human resource management.

### Health information management

**Fig. 1:**
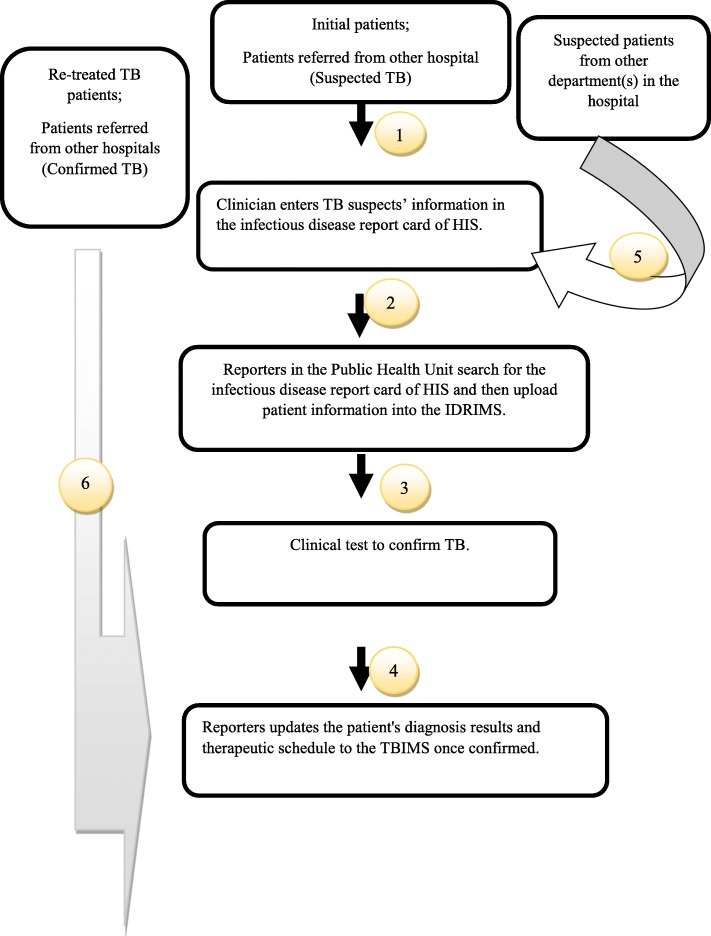
Procedure for Reporting TB, Fig. 1 outlines 6 main steps in the TB reporting process once a patient has been diagnosed with TB or suspected TB

Health information management consists of the administration of multiple steps in the reporting process, and the information management flow of the TB reporting system is very complicated. Through in-depth interviews and time spent performing the tasks of a TB reporter we have developed a figure to illustrate the information flow for reporting TB (Fig. 1).

As described in Fig. 1. the TB reporting system is complex. A TB suspect could be referred from other departments in the same designated hospital, other hospitals or primary health care (PHC) facilities. The first clinician to diagnose patients with suspected TB after a chest X-ray is responsible for completing the infectious disease report card in NNDRS (**Process 1 & 5)**. However, clinicians usually direct TB suspects to the TB clinic, and leave the TB clinician to fill in the infectious disease report card in NNDRS. TB clinicians in the designated hospitals do not have permission to check if a patient’s card was already entered in NNDRS by other hospitals. Therefore, when patients are referred from other medical facilities they are required to bring a referral sheet, which indicates if they have confirmed TB or not. If not, the TB clinician needs to fill in the infectious disease report card in NNDRS. Duplication occurs if that patient’s card had already been created as a TB suspect in NNDRS by other hospitals, and in this case the CDC is responsible for manually deleting the duplicated reporting cases. The infectious disease report card completed by TB clinicians in the designated hospital is stored in HIS on the hospital intranet (**Process 2)**. Then the reporters in the Public Health Unit search for the infectious disease report card on a daily basis and manually upload the information into the NNDRS on the national internet. Sputum smear tests, sputum culture tests and X-ray tests are usually conducted in designated hospitals to confirm TB. The clinical test results are sent to the TB clinic on paper and are stored electronically in the hospital laboratory information system (LIS) (**Process 3)**. Reporters will update the patient’s information of diagnostic tests in NNDRS after manually retrieving test results through the hospital internal network (**Process 4)**. Next, they need to click on a confirmation button and the patient information will be automatically transferred from NNDRS to TBIMS. Patients’ therapeutic schedule and tracking records are subsequently updated in TBIMS by CDC personnel. If the infectious disease report cards are created by other hospitals, the reporters will manually enter the information in TBIMS. For re-treated TB patients and confirmed TB patients referred from other hospitals, clinicians are not required to complete the infectious disease card and medical staff can skip steps 1,2,3 and 4. But reporters need to update their information in TBIMS (**Process 6)**.

The first medical institution to diagnose patients with suspected TB is responsible for reporting cases to NNDRS, but this responsibility is in fact often left to TB clinicians. The first hospital (usually the designated hospitals) to confirm the diagnosis is then responsible for updating diagnostic information in NNDRS and reporting cases to TBIMS. Other comprehensive hospitals may be able to diagnose TB patients but they do not have access to TBIMS, and they are required to refer TB suspects/patients to designated TB hospitals.

Based on our interviews, hospitals use different electronic systems to record patient information, which often leads to confusion when health staff responsible for reporting TB cases search the records for a TB diagnosis. For example, Hospital A’s HIS allows the diagnosis result section to be completed in narrative form, however the staff responsible for entering TB cases into the system may not fully understand the clinical narrative, depending on its complexity, which may lead to incorrect information being entered into NNDRS and TBIMS. By contrast, Hospital B’s HIS diagnosis result section cannot be completed in narrative form, and does not include an option to enter “suspected tuberculosis”. As a result, clinicians cannot enter information on suspected cases so leave the TB diagnosis section blank, however if and when a TB case has been confirmed they fail to go back to the system to change the diagnosis, resulting in an unreported TB case. These issues were highlighted during our interviews with hospital staff and are illustrated by the responses in the Additional file 2.

TB patients require a valid ID in order to be entered into the TBIMS. However, neither HIS nor NNDRS can automatically validate a patient’s ID number, so if an ID number is fake, or if it was incorrectly entered into HIS or IDRIMS, it cannot be transferred to the TBIMS and will ultimately go unreported.

Moreover, HIS, NNDRS, and TBIMS have no automatic method of identifying and removing duplicated TB cases. Instead, TB personnel are required to manually identify and delete duplications, a step that adds to the burden of an already time-consuming process. As a result, the individuals interviewed revealed that if they think they recognize a patient’s name they presume it is a duplication and do not enter it into the system in order to avoid having to manually remove it in the future. However, if the name is a new patient, their mistake results in an unreported case.

Another weak spot in the system is the lack of connection between the hospital intranet and extranet systems. For instance, in Process 2 (Fig. 1), HIS automatically reminds the clinician to complete an infectious disease report card, however, this information does not automatically transmit to the external network (NNDRS, TBIMS) but instead relies on TB reporters to manually transfer the information. Additionally, hospitals are free to choose their own software and operating systems for recording TB information. As a result, staff reported struggling to understand how to operate the different systems which could lead to data entry errors.

The health information system lacks interoperability among different health facilities. Hospital staff responsible for TB reporting can only access the systems in their own hospitals, so if a patient is transferred from another hospital, the hospital staff have to contact the referring hospital to get patient information. This slow, unautomated form of communication allows room for error. Duplication or missing records are caused if a patient’s infectious disease card either has either already been created and reported by the referring hospital and then reported again by the referral hospital or has not been created and reported by either the referring hospital or the referral hospital.

HIS is unable to automatically connect to TBIMS, as a result the reporting staff need to manually transfer and update this information. Further complication occurs during Process 6. Once lab results confirm a TB positive diagnosis, the staff responsible for reporting could misinterpret results and clinical notes and entering incorrect information in the system.

### Health human resource management

#### Health personnel/workload

Clinicians in the study hospitals have heavy workloads, and are reluctant to add to their duties with procedures related to TB reporting. Part of this reluctance may be caused by the fact that most clinicians regard diagnosis and treatment as their only responsibility and are not aware of the importance of timely TB reporting. Some clinicians disclosed during interviews that they deliberately entered new TB cases as re-retreated cases in order to avoid having to complete all the steps required for entering a new TB case within the reporting system (processes 2 and 6).

#### Incentives

Lack of financial incentive adds to the problems caused by the lack of sufficient knowledgeable health staff. Hospitals do not receive earmarked funds from the government to support the required TB reporting process, and health professionals are not well compensated for the extra duties required by the reporting process, resulting in a lack of enthusiasm and attention to detail.

#### Supervision and accountability

An important factor influencing all of the above issues is the lack of leadership and guidance provided at national and local levels in TB control. Chinese health facilities have a fair amount of autonomy (4) and lack accountability in complying with TB reporting procedures. Municipal or county- level CDCs are required to perform data quality checks, however, the CDCs at these levels are reluctant to do so, or are unable to do so effectively. This lack of oversight is due to lack of well-trained staff within the CDC and the fact that CDC officials often rank below the hospital staff they are supervising. This hierarchical system makes supervision difficult.

Lack of government oversight, unified reporting requirements and incentives during the establishment of the health information systems have all made the implementation of these systems more difficult.

These issues are illustrated by interview responses included in the Additional file 2.

## Discussion

Our study clearly indicates that a significant number of TB cases diagnosed in the hospitals were not accurately reported at the national level, as required by law. Similar findings have been found in other countries. Research on Indonesia found that its private health sector hardly reports TB cases, as required by its national health legislation (22). Similarly, there is also evidence that India, Namibia, and Thailand all suffer from a lack of national data standardization and accessibility (22–28), and might not accurately report the number of confirmed TB cases. It is also reasonable to assume that many other low- and middle-income countries without high functioning health information management systems could face similar problems. The underreporting of TB impacts the capacity to accurately evaluate the epidemiology of the disease. Therefore, a reliable TB surveillance and reporting system is essential for accurately mapping the disease and developing appropriate strategies to meet the WHO *End TB Strategy*.

Our study found two main areas contributing to underreporting: 1) management of human resources for health, and 2) health information management.

Our findings show that the heavy workload and poor incentives for health professionals responsible for reporting TB cases in the two Chinese hospitals contributed to the level of underreporting. Similar findings have been reported in Namibia, India and Bangladesh (22–24, 26, 27) where workforce shortages contribute to underreporting. Another contributing factor is the disjointed health information management system used to report TB cases in China. Likewise, in Namibia, a large number of systems, databases, and processes are manual and paper-based (24), and in India, private hospitals record data based on their own unique needs, rather than following the national reporting standards. Furthermore, the lack of accountability and adequate supervision evident in the Chinese hospitals are apparent in other countries. For instance, India has no independent authority to oversee data quality (23) and Bangladesh does not enforce data quality assurance and assessment systems (27, 29).

The second main factor associated with underreporting is the health information management system. Our study found that Chinese hospitals use different operating systems to record patient information and transfer information to the various TB reporting systems. By using different reporting systems, hospitals collect different information on patients and TB cases, making uniform data difficult to collect at a national level. Like China, the health information management systems in many low- and middle-income countries are fragmented and often include multiple vertical systems designed to meet the requirements of the national government or international organizations/donors. As a result, similar problems occur in other low and middle-income countries (2, 17, 22–27, 30). For example, in Indonesia, the reporting forms in each health center are different, which results in missing data and hampers the data standardization process (2, 17, 30).

Another weak spot in health information management system is the lack of accessibility to data. Our study found that in China, only high-level CDC staff can access TB data. This is problematic as lower level CDC personnel on the ground tracking TB cases cannot review the data if necessary. This is particularly hindersome in China where there are a high number of internal migrant workers, such hierarchical information systems make it difficult to trace the TB patients moving from one place to another especially as TB treatment regimens take many months to complete. In other low- and middle-income countries, accessing data is a common problem (22–27). In India, health institutions generate inconsistent and duplicated health data, and there is no comprehensive HIS to provide government offices and researchers with essential information for analyzing and surveilling data quality (22). In Thailand, data is collected by community health nurses on paper and then stored in cabinets. While some information is eventually entered into the government database, nurses and other healthcare workers cannot access it (25).

Overall, the reporting system relies on health personnel to enter data, keep it updated and correct using complicated and disjointed health information management systems. All of these factors leave room for errors and lead to global underreporting of TB.

## Limitations

This study is somewhat limited by the fact that only two hospitals in one region of Eastern China are included. However, this limitation is mitigated by the fact that the hospitals are located in a developed region with plenty of resources, therefore if hospitals with access to resources and relatively advanced information systems struggle with accurate reporting, it could be assumed that similar problems exist in regions and countries with fewer resources. We employed a strict inclusion criterion for selecting confirmed TB cases which excluded diagnosis records with any unclear marks or terminology which might undervalue the actual rate of underreporting. Lastly, due to the relatively small sample only a few independent variables were collected, making a multivariable analysis difficult. However, the qualitative data collected via interviews was used to explore a number of key factors associated with under-reporting.

## Conclusions

This study demonstrates that Eastern China, like other regions and countries with TB epidemics struggles with underreported TB cases. The factors associated with this problem include poor management of health professionals, and inadequate health information management. Similar problems may not be uncommon in other parts of China as well as in many low- and middle-income countries. Underreporting should be of high concern for both the global community and national government agencies responsible for TB control and care.

## Supplementary information

**Additional file 1.** Interview guide for semi-structured qualitative interviews. Complete interview guide translated into English used for semi-structured qualitative interviews with key stakeholders

**Additional file 2.** Interview Findings. Phrases from interviews that demonstrate qualitative findings

## Data Availability

The datasets used during the current study are available from the corresponding author on reasonable request.

## Abbreviations

CDC: Centers for Disease Control
HIS: Hospital Information System
NNDRS: National Notifiable Disease Reporting System
SARS: severe acute respiratory syndrome
SDG: Sustainable Development Goals
TB: Tuberculosis
TBIMS: TB Information Management System
WHO: The World Health Organization

## Footnotes

1 The names of city and hospitals were not used to in order to protect anonymity
