# Supplementary material for: Under-reporting of TB cases and associated factors: a case study in China
Source: BMC Public Health. 2019 Dec 11;19:1664. doi: 10.1186/s12889-019-8009-1 (PMC6907198; doi:10.1186/s12889-019-8009-1)
Supplement: Supplementary file 1 — Additional file 1. Interview guide for semi-structured qualitative interviews. Complete interview guide translated into English used for semi-structured qualitative interviews with key stakeholders [file 12889_2019_8009_MOESM1_ESM.docx]

**Interview Guide for In-Depth Interviews**

**Interview Outline for Health Information System**

**CDC:**

1. **The head of Zhenjiang CDC**

**Purpose:**

1) To know about the use of TBIMS and the supporting measures in Zhenjiang CDC.

2) To know the opinion of the head of Zhenjiang CDC on the use of TBIMS

3) To know problems on management of TB patients in TBIMS and his suggestions.

**Interview Outline:**

1) Introduce the use of TBIMS in the CDC.

A. Report: what is the reporting standard and reporting process of designated hospitals and non-designated hospitals? How does the CDC handle the reported data?

B. Analysis and its implication: will the data be analyzed? Which index are generally required? What is the implication of the analysis? Is the reporting unit aware of the analysis implication?

C. Quality check: Does anybody assess the quality of the collected data?? If so, who? How to assess? How to check it?

D. Information exchange: Do you have any information feedback?

Yes:

1. Which institutions and what mechanisms?

No:

1. Does anybody contact the hospital for further verification if something is wrong about the data?
2. Do you think is it necessary to provide feedback? What are the reasons or obstacles of failing to provide feedback?

2) How many people are responsible for the management of TBIMS in CDC? What are the responsibilities? Is there any special training? Do you have any regular performance appraisal and quality assessment?

3) How much fund do you have for TB related management? Where do you mainly spend that fund? Is the fund sufficient?

4) What policies on management of TB patients do you have in CDC?(How to manage the patients from different districts, how to manage the floating patients?) What is the status quo of implementation

5) How municipal CDC cooperate with the designated hospitals, county and district CDC, grass-roots health institutions under the TBIMS and the IDRIMS?

6) Please provide a comprehensive evaluation of TBIMS.

A. Does the data processing, collection and analysis meet the management requirements of TB patients? Is it convenient for epidemic surveillance and patient tracking (especially for immigrants and referral patients)?

B. What is the satisfaction rate of the TBIMS? (system operation, data analysis, patient management, communication with institutions, etc.)

C. Is there any missing report or data mismatch in the system? How often? What is the most possible reason according to your knowledge?

D. What challenges do you still face?

7) What are your opinions and suggestions on the information system to meet the needs of reporting and managing patients of infectious diseases?

1. **Reporter of Zhenjiang CDC TBIMS**

**Purpose:**

1) To know the quality of collected data in the TBIMS and the challenges of daily use.

2) To know the opinions and evaluations of the Special Reporter in Zhenjiang CDC on the TBIMS.

3) To know the Special Reporter's suggestions on the improvement of the TBIMS.

**Interview Outline:**

1) Briefly introduce your responsibility in TB data management.

a. How long have you been in this job? How long is your pre-job training? Will the CDC assess the quality regularly?

B. How to deal with data of TB patients uploaded from hospitals or grass-root health institutions?

C. How much time has been spent on the TBIMS? What is the workload?

D. How do you deal with non-local TB patients in general? Do you contact CDC in their location?

E. Are you satisfied with the current job? (Workload, compensation, etc.)

2) Do you assess the quality of the collected data? If so, who? How to assess? How to check it?

A. Is there any information feedback?

Yes:

1. Which institutions and what mechanisms?

No:

1. Does anybody contact the hospital for further verification if something is wrong about the data?
2. Do you think is it necessary to provide feedback? What are the reasons or obstacles of failing to provide feedback?

B. How CDC reacts if something is wrong about the data? Does anybody take the responsibility?

3) Please provide a comprehensive evaluation on the TBIMS.

a. Is the interface user-friendly and easy to understand? Is the system fully functional logically?

B. Does the data processing, collection and analysis meet the management requirements of TB patients? Is it convenient for epidemic surveillance and patient tracking (especially for immigrants and referral patients)?

C. What is your satisfaction rate of the TBIMS? (system operation, data analysis, patient management, communication with agencies, etc.)

D. Is there any missing report or data mismatch in the system? How often? What is the most possible reason according to your knowledge?

E. What are your difficulties in daily operation? How to solve it?

4) What are your opinions and suggestions on the information system to meet the needs of reporting and managing patients of infectious diseases?

**Designated Hospitals:**

1. **Leaders who are responsible for infectious diseases in designated hospitals**

**Purpose**

1) To know the status and policies of TB management in designated hospitals

2) To know his opinions, evaluation and suggestions on HIS and TBIMS

**Interview Outline**

1) Please give a brief introduction on the status quo of TB patients reporting in hospital.

A. Who is the currently responsible for registering TB patient data and upload it to TBIMS? Is this person full-time? How long is the training? Who is the trainer?

B. How do you refer patients inside the hospital if you find TB patients in different departments of the hospital? Is the referral rate high currently? Does the hospital have any special policies for referral of TB patients in non-infectious departments?Are clinicians in non-infectious departments trained in reporting infectious diseases?

C. How to manage and report non-local patients, duplicate smear-negative TB patients, recurrent patients, outpatient patients and in-hospital referral patients? (Go down the details here)

D. What is the investment in TB control in the hospital? Is there any support for special reporter? Is there any person responsible for information system construction and maintenance? (How important is this in the hospital?)

2) Please introduce the usage of hospital information system.

A. System joint: Is the HIS joint with other system like TBIMS, IDRIMS?

If it is not joint:

1. Do you want it to have such feature? What improvements will it bring to your work after they joint? What are the possible difficulties?

B. Information exchange: Is there any information feedback?

Yes:

1. Which institutions and what mechanisms? (It is required to mention the non designated hospitals which refer the patients)

No:

1. Do you contact that institution for further verification if something is wrong with data?
2. Do you think is it necessary to provide feedback? What are the reasons or obstacles of failing to provide feedback?

3) Please introduce the treatment management and expenses of TB patients.

A. Do the health administration departments or medical insurance policy have limits on hospitalized days, total expenses and self owned expenses for TB patients?

B. Do you have any special payment methods for TB patients? (prepaid in lump sum? Pay by project? ）

C. Is there any other health departments or health insurance policy that may affect information management of TB patients?

4) Please provide a comprehensive evaluation of the information system in hospital.

A. Does the data processing, collection and analysis meet the management requirements of TB patients, especially for migrants and referrals?

B. Does the information system meet the requirements of the Infectious Diseases Prevention Law?

C. What is your satisfaction rate of the information system in hospital? (System operation, data input, analysis, reporting, patient management, communication with other departments, communication with other institutions, etc.)

D. Is there any missing report or data mismatch in the system? How often? What is the most possible reason according to your knowledge?

E. What challenges do you still face?

5) How to cooperate with CDC and grass-root health institutions under the information system?

6) What are your opinions and suggestions on the information system to meet the needs of reporting and managing patients of infectious diseases?

1. **TB Reporter of Designated Hospital**

**Purpose**

1) To know the status quo of the operation of the information system and the standards and procedures for uploading data in designated hospital.

2) To know the quality of reporting data and the possible reason of missing reports in designated hospitals.

3) To know the opinions, evaluation and suggestions of designated hospitals on HIS and TBIMS

**Interview Outline**

1) Please briefly introduce your work.

A. How do you deal with reported data of TB patients from various departments?

B. How long have you been in this job and how long is your pre-job training? Will the hospital conduct performance appraisal regularly?

C. How frequent is the transfer of staff in disease control department? Is it frequent? (If it is frequent, how can you guarantee the quality?)

D. How do you think of your workload?

E. Are you satisfied with the current job? (Workload, compensation, etc.)

2) What are the standards and procedures for uploading information of TB patients and their medical records in designated hospitals under the information system?

A. What is the standard of reporting patients? (suspected cases, floating population, outpatient patients, referral patients in hospital, duplication smear negative patients, TB pleurisy patients, etc.)

B. What is the procedure of reporting patients? ↑

C. Is the HIS joint with other information systems (such as TBIMS and IDRIMS)?

Yes:

1. Do the records upload to the disease control department automatically? Or Does the disease control department automatically grab the records of TB patients in HIS and report?

No:

1. Do you want it to have the feature of automatic uploading in the future? What improvements will it bring to your work? What are the possible difficulties?

D. Does the HIS push notification to you?

E. Does the staff in the department of Prophylaxis have access to the information of the entire hospital? (if the other party is confused, explain the purpose of the question to him, such as unable to check the data if something is wrong in the data, etc.)

3) Do you access the quality of the collected data? If so, who? How to assess? If so, how to check it? (Will you check the data quality for TBIMS? Will check it by comparison with HIS? )

A. Is there any information feedback?

Yes:

1. Which institutions and what mechanisms? (It is required to mention the non designated hospitals which refer the patients)
2. Is there any channel to provide the feedback to various departments? Will you contact the doctor when there is logical mistake?

No:

1. Do you contact that institution for further verification if something is wrong with data?

2. Do you think is it necessary to provide feedback? What are the reasons or obstacles of failing to provide feedback?

B. How to deal with errors and missing in data (such as missing ID)? （Is there any possibility to delete the error record manually?）

C. Is there any mechanism to avoid quality problems such as missing, duplicate report in the information system? Is there an automatic logic check?

D. Who shall be accountable for the quality problem in data? Are there any rewards and punishments for reporting TB information?

4) Please provide a comprehensive evaluation of reporting system in the hospital.

A. Is the interface user-friendly and easy to understand? Is the system fully functional logically? Do you have any difficulties in daily operation, such as confused functions of the system, operation failures, etc.? How to solve it?

B. Is it easy to meet the format requirement when uploading data? What is the amount of narrative answers, does it increase the workload?

C. Does the data processing, collection and analysis meet the management requirements of TB patients, especially for migrants and referrals from other hospital and inside the hospital?

D. What is your satisfaction rate of the TBIMS? (System operation, data input, analysis, reporting, patient management, communication with other departments, communication with other institutions, etc.)

E. Is there any missing report or data mismatch in the system? How often? What is the most possible reason according to your knowledge?

F. What are your difficulties in daily operation? How to solve it?

5) What are your opinions and suggestions on the information system to meet the needs of reporting and managing patients of infectious diseases?

1. **TB clinicians in designated hospitals**

**Interview Outline**

1) How to contact the reporter of TBIMS after receiving TB patients?

A. Please indicate the differences among the outpatients, inpatients, non-local patients and referred patients? What is the specific process? What information should you provide to him?

B. Are you willing to see that the HIS is joint with TBIMS in the hospital? What improvements will it bring to your work? What are the possible difficulties?

2) Does the TB reporter contact clinicians regularly to check the data quality of TB patients in HIS? If so, how to check it?

A. Is there any channels to provide feedback among various departments? How to cooperate with different departments? Will you actively remind and communicate about TB patients?

B. Will you contact the non designated hospitals in which the referred patient was to confirm the information?

C. Who shall be accountable for the quality problem in data? Are there any rewards and punishments for reporting TB information?

D. Is there an infection surveillance team in the hospital to supervise the quality? How about the quality assessment about monthly missing report and infection control in hospital?

E. What do you think is the most possible reason for data mismatch?

3) What else do you want to say about the reporting process of TB patients?

4) What are your opinions and suggestions on the information system to meet the needs of reporting and managing patients of infectious diseases?
