# Supplementary material for: Under-reporting of TB cases and associated factors: a case study in China
Source: BMC Public Health. 2019 Dec 11;19:1664. doi: 10.1186/s12889-019-8009-1 (PMC6907198; doi:10.1186/s12889-019-8009-1)
Supplement: Supplementary file 2 — Additional file 2. Interview Findings. Phrases from interviews that demonstrate qualitative findings [file 12889_2019_8009_MOESM2_ESM.docx]

**Interview Responses on the Difficulty in Information Flow Management**

“Some clinicians like to use their own marks to write the diagnosis results. For example, they recorded the retreated TB patients as ‘fz’ (FuZhen, is the abbreviation of Chinese of “retreated”), and they usually recorded the suspected TB patients as ‘TB-’ or ‘TB?’, which is very difficult for us to find the patients out in the hospital intranet through our search engine. And sometimes it is confusing for us to determine if the TB patients should be reported or not…” （Reporting staff 1; Hospital A）

“I hope our HIS can include the option of ‘suspected tuberculosis’, or at least add a remark to remind me that this person still needs a further smear examination. Sometimes the clinician calls me to tell me not to report the patient in TBIMS because the patient hasn’t received all the three smear tests yet. But sometimes I am busy… so after the patient is confirmed you know …”. (Reporting staff 3; Hospital B)

“I will only report the information that I find within our hospital intranet. If I am confused with something, I contact the clinician through WeChat …” (Reporting staff 1; Hospital A)

“I only have the authority to check the patients’ information reported from our hospital.” (Reporting staff 1, Reporting staff 2, Hospital A; Reporting staff 3, Hospital B)

“The patients referred from other hospitals bring a referring sheet, which indicates whether or not they have been reported in TBIMS. If they are confirmed TB patients, we usually believe that they have been reported by their previous hospitals. Nonetheless, we have no authority to check if they indeed have been reported in TBIMS.” (Reporting staff 3, Hospital B)

**Interview Responses on Health Personnel, Incentives and Supervision and Accountability**

“TB reporting? No, no one has contacted me before. My duty is just to fill in the infectious disease card, that’s all. Reporting is someone else’s duty, I don’t know what happens in other departments.” (Clinician 1; Hospital A)

“Besides reporting TB cases, my daily work includes many other things, such as reporting all the infectious diseases, vaccinations, giving primary care … sometimes I feel I am too busy to die, it depends on how many patients come here today. (Reporting staff 3; Hospital B)

“If the clinician clicks the ‘re-treated patient’ button to skip the process of filling in the infectious disease card, no one in the hospital can find out if the patient has been reported in TBIMS or not unless the CDC personnel come to check our underreporting rate … If you ask me whether I have done the same thing before, of course not. But I would say that others may have done so …” (Clinician 2; Hospital A)

“Recently our hospital leader took the issue of underreporting very seriously. He often holds conferences to emphasize the importance of timely reporting for infectious diseases. But that’s all.” (Clinician 3, Clinician 4; Hospital B)

“You see, every day I need to diagnose and treat TB patients, I am very busy. You cannot expect me to check the accuracy of the reported information. It’s not my duty. It’s the reporter’s duty.” (Clinician 1; Hospital A)

“My work includes reporting and checking the TB patients’ information, writing monthly review of TB epidemiology, tracing patients, visiting each patient’s home and reminding them to take medicines …The only TB personnel here is me. It’s absolutely impossible for me to spend too much time dealing with the patients who are difficult to trace.” (Reporting staff 5; CDC)

“Although we do have a regulation that a missing case will lead to a decrease in bonus for related personnel, as it is difficult to trace the individual responsible, this regulation has never been implemented. And in a small prefecture, the CDC has a good relationship with the hospitals, we are all acquaintances. So, it’s difficult for us to penalize certain clinicians or reporters ……” （Leader 1; CDC）

“I really dislike filling in the infectious disease report cards. It’s so time-consuming and I get no extra money.” (Clinician 1; Hospital A)

“I should have 50 Yuan (about US$ 7.3) per patient as a bonus to manage and trace TB patients. But I never see it. Maybe it is already included in my salary? But my salary is so low. And I think it’s unfair that my heavy work load is only worth 50 Yuan.” (Reporting staff 5; CDC)

“Actually, the government funds for TB tends to go into the whole pool of total funds of the local CDC.TB prevention is important, but HIV, Hepatitis BC prevention is also important. And since the standard of subsidy for the TB specialist is not clear, we tend to allocate some TB funding to other infectious disease prevention projects.” (Leader 1; CDC)

“There are no detailed standards and indicators for us to follow. The upper-level CDC only requires us to decrease the TB epidemiology, to decrease the TB underreporting rate. But they didn’t tell us how to reach the goals and to what extent we should achieve the goals. We are really confused about it. The same confusion also happens to the provincial CDC, they don’t know how to oversee and guide us as well. So, in practice, unless the central government clearly tell us that they would come to investigate our working quality and check certain statistic rates, we are in the situation that ‘we do work, we oversee ourselves’.” (Leader 1; CDC)
